# Bacterial burden in the lower airways predicts disease progression in idiopathic pulmonary fibrosis and is independent of radiological disease extent

**DOI:** 10.1183/13993003.01519-2019

**Published:** 2020-04-02

**Authors:** Rachele Invernizzi, Joseph Barnett, Bhavin Rawal, Arjun Nair, Poonam Ghai, Shaun Kingston, Felix Chua, Zhe Wu, Athol U. Wells, Elizabeth R. Renzoni, Andrew G. Nicholson, Alexandra Rice, Clare M. Lloyd, Adam J. Byrne, Toby M. Maher, Anand Devaraj, Philip L. Molyneaux

**Affiliations:** 1 National Heart and Lung Institute, Imperial College London, London, UK; 2 Royal Brompton Hospital, London, UK; 3 Dept of Radiology, University College Hospital, London, UK; 4 Contributed equally as first authors

## Abstract

Increasing bacterial burden in the lower airways of patients with idiopathic pulmonary fibrosis confers an increased risk of disease progression and mortality. However, it remains unclear whether this increased bacterial burden directly influences progression of fibrosis or simply reflects the magnitude of the underlying disease extent or severity.

We prospectively recruited 193 patients who underwent bronchoscopy and received a multidisciplinary diagnosis of idiopathic pulmonary fibrosis. Quantification of the total bacterial burden in bronchoalveolar lavage fluid was performed by 16S rRNA gene qPCR. Imaging was independently evaluated by two readers assigning quantitative scores for extent, severity and topography of radiographic changes and relationship of these features with bacterial burden was assessed.

Increased bacterial burden significantly associated with disease progression (HR 2.1; 95% CI 1.287–3.474; p=0.0028). Multivariate stepwise regression demonstrated no relationship between bacterial burden and radiological features or extent of disease. When specifically considering patients with definite or probable usual interstitial pneumonia there was no difference in bacterial burden between these two groups. Despite a postulated association between pleuroparenchymal fibroelastosis and clinical infection, there was no relationship between either the presence or extent of pleuroparenchymal fibroelastosis and bacterial burden.

We demonstrate that bacterial burden in the lower airways is not simply secondary to the extent of the underlying architectural destruction of the lung parenchyma seen in idiopathic pulmonary fibrosis. The independent nature of this association supports a relationship with the underlying pathogenic mechanisms and highlights the urgent need for functional studies.

## Abstract 2

**Elevated bacterial burden predicts mortality in IPF and is independent of both radiological features and extent of disease** http://bit.ly/2RaDbdv

## Introduction

The incidence of idiopathic pulmonary fibrosis (IPF) continues to rise as does the burden of related mortality [1]. Whilst a precise understanding of the pathogenesis remains elusive, there is a growing appreciation of the importance played by the interaction between the lung and environment in the development of IPF [2–4]. Over the past decade, our understanding of the dynamic and complex bacterial communities in the lower airways (the microbiome) and their role both in health and disease has increased dramatically [5]. Although, historically, infection was not considered a major driver in IPF it is now clear that infective episodes carry the same devastating mortality as acute exacerbations of the disease [3, 6]. Furthermore, even at diagnosis and in the absence of infection there is a dramatic increase in bacterial load with altered composition of the respiratory microbiome in the lower airways of patients with IPF [7, 8].

We and others have highlighted differences in microbiome diversity and levels of individual bacteria when comparing both healthy individuals and those with IPF and when comparing individuals with IPF who are stable or progressive. A number of associations between components of the microbial communities and cytokines, peripheral blood-transcriptomic profile and disease behaviour have been identified [9, 10]. More recently, animal models have begun to tease out mechanisms by which dysbiosis in the lower airways can drive fibrosis [11]. The best validated microbial signal identified in IPF is that of the lower airway bacterial burden. At the time of diagnosis, individuals with IPF have a higher bacterial burden than healthy individuals and subjects with chronic obstructive pulmonary disease. Importantly, across IPF subjects, the level of bacterial burden relates to survival and increases further during culture-negative acute exacerbations [12].

It remains unclear whether bacterial burden directly influences progression of fibrosis or if bacterial numbers instead reflect other underlying disease processes. While previous studies have accounted for disease severity using physiological parameters (including forced vital capacity (FVC) and measures of gas transfer (diffusing capacity of the lung for CO (*D*_LCO_)), there has been no assessment of the relationship between bacterial burden and radiological markers of fibrotic distortion of the lung or disease extent or severity. We hypothesised that if the bacterial burden is elevated secondary to the underlying architectural destruction of the lung parenchyma then there might be a clear association between radiographic findings such as honeycombing or traction bronchiectasis and bacterial burden in the lower airways of individuals with IPF.

## Methods

### Patient recruitment

Patients undergoing diagnostic bronchoscopy with bronchoalveolar lavage (BAL) for suspected IPF were prospectively recruited between November 2015 and January 2017. Only subjects receiving a multidisciplinary team diagnosis (MDT) of IPF according to current American Thoracic Society (ATS)/European Respiratory Society (ERS)/Japanese Respiratory Society (JRS)/ Latin American Thoracic Association (ALAT) guideline definitions were included [13]. Subjects were excluded if they had a history of self-reported upper or lower respiratory tract infection, antibiotic use in the prior 3 months, acute exacerbation of IPF, or other respiratory disorders. Written informed consent was obtained from all subjects, and the study was approved by the local Research Ethics Committee (10/HO720/12 and 15/SC/0101).

### Bronchoscopy

Fibreoptic bronchoscopy with BAL was performed *via* the oropharyngeal route as previously described [7]. Briefly, 60-mL aliquots of warmed saline, to a total volume of 240 mL, were separately instilled into a segment of the right middle lobe. Post-collection, an aliquot of unfiltered and unprocessed BAL was immediately placed on ice, snap frozen and stored at −80°C. Negative control samples were collected by aspirating buffered saline through the bronchoscope suction channel before use. Cell differentials of macrophages, lymphocytes, neutrophils and eosinophils were performed as previously described [14].

### Radiology

The CT variable definitions were based on the Fleischner Glossary of terms [15] and current ATS/ERS/JRS/ALAT guideline definitions [13]. Computed tomography (CT) data were scored using the following scales: to the nearest 5% per lobe (fibrosis, honeycombing), on a binary basis (usual interstitial pneumonia (UIP)), on a lobar extent with the lingua defined as the sixth lobe (pleuroparenchymal fibroelastosis (PPFE)) [16] and on an 18-point scale (traction bronchiectasis) [17].

### Bacterial DNA extraction

BAL samples (2 mL) were centrifuged at 21 000×*g* for 30 min to pellet cell debris and bacteria. Pellets were resuspended in 100 μL of supernatant and added to lysing matrix E tubes (MP Biomedicals, Solon, OH, USA) containing 500 μL cetyl trimethylammonium bromide (CTAB) buffer (10% w/v CTAB in 0.5 M phosphate buffered NaCl) and 500 μL phenol:chloroform:isoamyl alcohol (25:24:1), and shaken in a FastPrep Instrument (MP Biomedicals) at 5.5 ms^−1^ for 60 s. Following bead-beating, samples were extracted with an equal volume of chloroform:isoamyl alcohol (24:1), DNA was precipitated with 2 volumes of precipitation solution (30% w/v PEG6000 in NaCl) and, following ethanol washing, was resuspended in 100 μL Tris-EDTA. The quality and quantity of the isolated DNA was measured using a NanoDrop 1000 Spectrophotometer (Thermo Fisher Scientific, Hemel Hempstead, UK) and the DNA was stored at −80°C until further use.

### 16S rRNA gene quantitative PCR

Triplicate 10 μL quantitative PCRs (qPCR) were set up, containing 1 μL of bacterial DNA and 9 μL of Femto bacterial qPCR premix (Cambridge bioscience, Cambridge, UK). Each run contained a 10-fold dilution series of the *Vibrio natriegens DSM 759* gene cloned into a plasmid of known size and a non-template control. For data acquisition, the following cycling parameters were used: 1 cycle of 95°C for 10 min; 40 cycles of 95°C for 30 s, 50°C for 30 s, 72°C for 1 min; and 1 cycle of 72°C for 7 min [18].

### Statistical analysis

Continuous variables are presented as mean±sd and categorical variables as proportions. The time-to-event curves were calculated using the Kaplan–Meier method and compared with the use of the log-rank test. Differences between subject groups were evaluated with the use of the Mann–Whitney test for continuous variables and Fisher's exact test for categorical variables. Spearman's *rho* was used to calculate correlations between continuous variables. Assessment for collinearity of predictor variables was made using correlation coefficients. Bacterial burden data were found to be skewed and were log transformed. Transformed bacterial burden data were examined using univariate and stepwise multivariate linear regression models against CT features and cell differentials. All analyses were performed with the use of R [19]. A two-sided p-value <0.05 was considered to indicate statistical significance.

## Results

### Subjects and sampling

Between 2014 and 2017, 193 subjects undergoing diagnostic bronchoscopy, resulting in a final MDT of IPF, were prospectively recruited. 19 subjects underwent cryo- or surgical lung biopsy and this information was available to the MDT when assigning a diagnosis. Reflecting the demographics of IPF, the cohort was predominantly male, with an average age of 70 years and a significant smoking history (table 1). Of the 193 patients included in this study, 107 had concomitant gastro-oesophageal reflux (GORD) (defined as either self-reported symptoms or asymptomatic use of proton pump inhibitors (PPI)). 97 subjects in total were on anti-reflux therapy. 36 subjects were receiving inhaled corticosteroids at the time of bronchoscopy and 21 were on oral corticosteroids (<10 mg·day^−1^) (supplementary table S1).

**TABLE 1:** Clinical and radiological characteristics of patients with idiopathic pulmonary fibrosis

**Demographics**	**N=193**
**Sex male**	140 (73)
**Age years**	70±8
**Smoking status**	
Never	70 (36)
Ex	113 (59)
Current	10 (5)
**Pulmonary function tests**	
FVC % predicted	79±18
*D*_LCO_ % predicted	48±14
**Radiology**	
UIP probable/definite	57.7
Honeycombing	30
PPFE	21

Data are presented as n (%), mean±sd or %. Radiological variables are expressed as presence or absence. FVC: forced vital capacity; *D*_LCO_: diffusing capacity of the lung for CO; UIP: usual interstitial pneumonia; PPFE: pleuroparenchymal fibroelastosis.

### Bacterial burden predicts mortality in IPF

**FIGURE 1:**
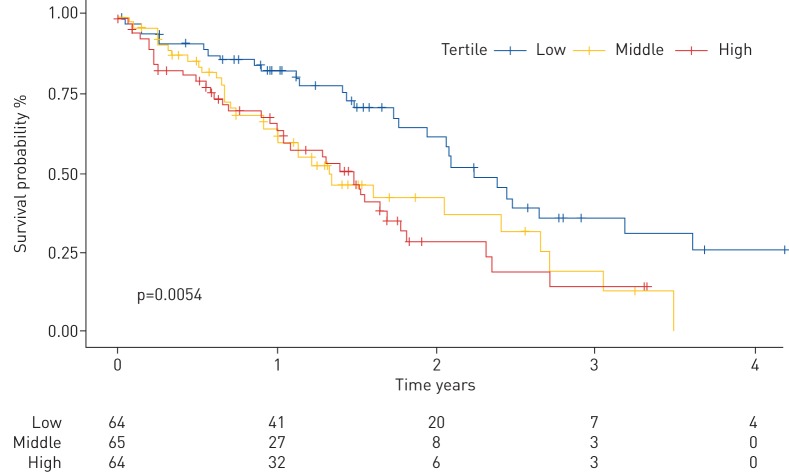
Increased bacterial burden in idiopathic pulmonary fibrosis at the time of diagnosis increases mortality. Kaplan–Meier curve generated by Cox proportional-hazards model stratified by bacterial burden (low tertile: 4.15×10^3^–3.17×10^4^ 16S rRNA gene copies·mL^−1^ of bronchoalveolar lavage (BAL) fluid; middle tertile: 3.33×10^4^–1.90×10^5^ 16S rRNA gene copies·mL^−1^ of BAL fluid; high tertile: 1.90×10^5^–5.44×10^6^ 16S rRNA gene copies·mL^−1^ BAL fluid). Log rank p test value reported.

Bacterial DNA was successfully extracted from all subjects and quantified using qPCR. The mean bacterial burden was 2.87×10^5^ (±1.63×10^6^) 16S rRNA gene copies per mL of BAL. This is elevated compared to historical controls and similar to previously published burdens [11]. Technical controls (saline flushed through the working channel of the bronchoscope), as anticipated, demonstrated counts close to or below the lower limit of qPCR quantification (100 copies·mL^−1^) (supplementary figure S1). To confirm the association of bacterial burden with disease progression, a composite end point of all-cause mortality within the first year or a decline of 10% or more in FVC at 12 months was employed. A total of 102 patients fulfilled the criteria for progressive disease. Using an unadjusted Cox proportional hazards model, bacterial burden significantly associated with disease progression (HR 1.20; 95% CI 1.06–1.35; p=0.0034). This association remained significant after adjustment for age, sex, baseline % predicted FVC, baseline % predicted *D*_LCO_ and smoking status in a multivariable Cox proportional hazards model (HR 1.16; 95% CI 1.02–1.30; p=0.0022). A survival analysis confirmed that subjects in the tertile with the highest bacterial burden had a significantly increased hazard compared with those in the lowest tertile (HR 2.1; 95% CI 1.287–3.474; p=0.0028). Moreover, individuals in the middle tertile were also at a substantially increased risk of mortality compared with subjects in the tertile with the lowest bacterial burden (HR 2.0; 95% CI 1.197–3.307; p=0.008) (figure 1). There were no significant differences in age, sex, smoking history or disease severity between the patients with IPF within these tertiles of bacterial burden.

### Bacterial burden and BAL differential cell counts

**FIGURE 2:**
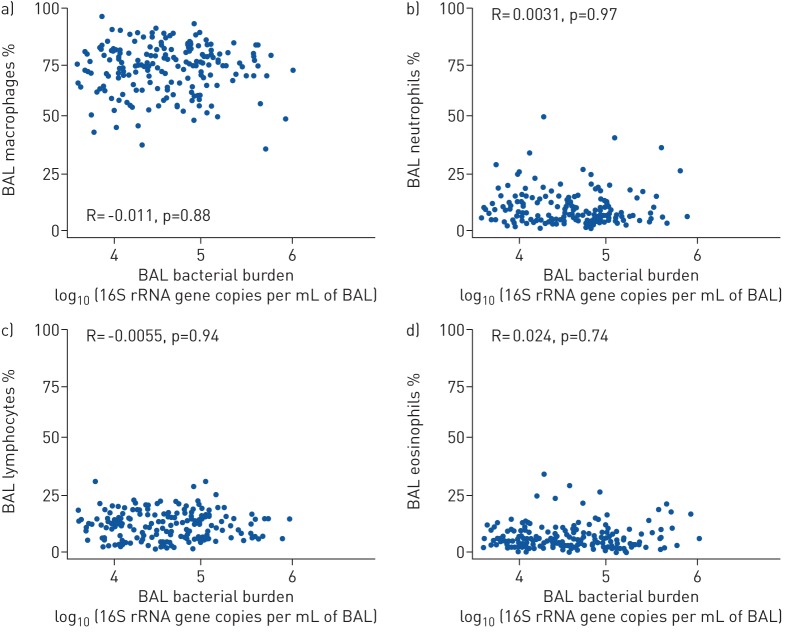
No correlation between bronchoalveolar lavage (BAL) differential cell counts and bacterial burden of subjects with idiopathic pulmonary fibrosis (IPF). Illustrating correlation between bacterial burden and percentage of (a) macrophages, (b) neutrophils, (c) lymphocytes and (d) eosinophils in BAL of subjects with IPF (n=193). Bacterial burden calculated by qPCR and expressed as log10 16S rRNA gene copies·mL^−1^ of BAL.

Next, the relationship between BAL differential cell count and bacterial burden was examined. As anticipated, the BAL cell differential for this cohort of individuals with IPF showed a predominance of alveolar macrophages 71.3±10.86% and neutrophils 9.86±7.78% (supplementary figure S2). A linear model was used to assess for any association between bacterial burden and BAL inflammatory differential cell count (supplementary table S2). Interestingly, there was no association (Spearman's rho: −0.093, p=0.24) between bacterial burden and total numbers of leukocytes per mL of BAL fluid (supplementary figure S3) or the proportion of any specific airway-inflammatory cell type (figure 2).

### Bacterial burden and physiological disease severity

We then sought to investigate whether there was a relationship between baseline disease severity, measured by FVC (% predicted) and *D*_LCO_ (% predicted), and bacterial burden. However, no association (FVC, % predicted: Spearman's rho: −0.013, p=0.86; *D*_LCO_, % predicted: Spearman's rho: −0.002 p=0.98) was found between the two (supplementary figure S4). While we did not set out to address the issues of GORD or concomitant medication use directly, we saw no effect on the bacterial burden based on the use of low-dose corticosteroids, inhaled steroids or PPI.

### Bacterial burden and radiological disease extent

**FIGURE 3:**
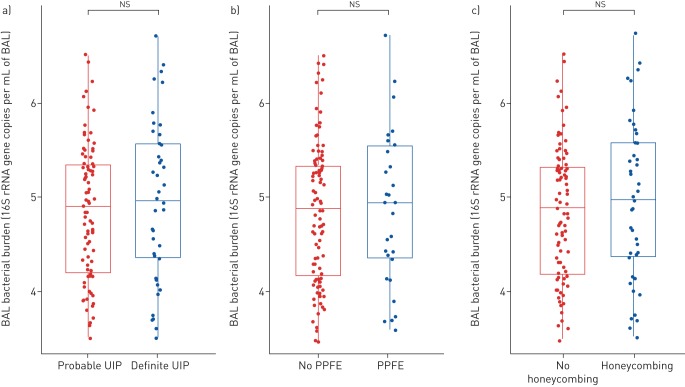
Relationship between radiological features and bronchoalveolar lavage (BAL) bacterial burden. No difference in bacterial burden log10 (16S rRNA gene copies·mL^−1^ of BAL) based on (a) a definite or probable pattern of usual interstitial pneumonia (UIP), (b) the presence or absence of radiological pleuroparenchymal fibroelastosis (PPFE) or (c) the presence or absence of honeycombing. Statistical significance tested with Mann–Whitney test.

Of the overall cohort, 140 subjects had CT imaging performed within 4 months of bronchoscopy. The median (range) time from bronchoscopy to imaging was −31 (−117–120) days. CTs were scored by two independent radiologists [A. Nair, B. Rawal], blinded to all clinical data. In cases of disagreement >20% between radiologists (incorporating >2 lobe difference in lobar scores, traction bronchiectasis score of >3 or disagreement of a binary variable), data were arbitrated by a third radiologist [A. Devaraj]. For scores evaluated to the nearest 5%, the whole lung score was defined as the mean lobar score. The mean score between radiologists, following arbitration, was used as the final score for analysis. In general, there was a reasonable agreement between radiologists (supplementary table S3) [20, 21]. Over half of the cohort had probable or definite UIP (57.7%) according to the current ATS/ERS/JRS/ALAT guidelines [13]. 21% (30 out of 141) of subjects had radiological evidence of PPFE and 30% (43 out of 141) had honeycombing (table 1). The relationship between radiological markers of disease severity and bacterial burden was explored using multivariate stepwise regression. CT fibrosis extent and CT traction bronchiectasis score were strongly collinear (Spearman's rho: 0.57), and so were included in separate models. There was no association between bacterial burden and radiological extent of disease or extent of either traction bronchiectasis or honeycombing (table 2). When specifically considering patients with definite or probable UIP, there was no difference in bacterial burden between these two groups (figure 3a). Furthermore, no difference in bacterial burden was observed when comparing IPF patients with either indeterminate or probable/definite UIP (supplementary figure S5). Despite a postulated association between PPFE and clinical infection [22], there was no relationship between either the presence or extent of PPFE and bacterial burden (figure 3b). Furthermore, no difference in bacterial burden was found when considering the presence or absence of honeycombing (figure 3c).

**TABLE 2:** Radiological features and their univariate prediction of bronchoalveolar lavage bacterial burden

	**Value**	**β estimate**	**se**	**Adjusted p-value**
**Traction bronchiectasis extent score**	6.7±3.0	0.691	1.750	0.492
**Fibrosis extent %**	22.0±10.7	1.050	0.921	0.297
**Honeycombing extent %**	2.6±6.8	−0.028	1.830	0.978
**Definite or probable UIP pattern**	88.0 (62.4)	−0.597	0.278	0.552
**PPFE lobar extent**	0.4±0.9	0.414	1.140	0.680

Data are presented as mean±sd or n (%), unless otherwise stated. Traction bronchiectasis scored on an 18-point scale. PPFE scored by number of lobes involved. Univariate regression performed against log transformed 16S rRNA gene copy number. se: standard error; UIP: usual interstitial pneumonia; PPFE: pleuroparenchymal fibroelastosis.

We then incorporated radiographic features of disease severity into the survival model to assess if bacterial burden predicts survival independent of radiographic features. Bacterial burden remained an independent predictor of survival even when incorporating radiographic features into the survival models and following adjustment for age, sex, baseline % predicted FVC, baseline % predicted *D*_LCO_ and smoking status. Again, given that CT fibrosis extent and CT traction bronchiectasis score were strongly collinear (Spearman's rho: 0.57), they were included in separate models. In both models, bacterial burden remained predictive of disease progression (HR 1.16; 95% CI 1.01–1.35; p=0.04).

## Discussion

This study validates previous findings that, for individuals with IPF, an increased bacterial burden at the time of diagnosis confers a worse survival. However, we demonstrate that this increased bacterial burden is independent of radiological markers of disease morphology and severity. This work, the largest prospective study of bacteria in the lower airways of individuals with IPF performed to date, confirms the importance of airway bacterial burden in IPF. Furthermore, the lack of association seen with measures of disease severity and fibrotic destruction of the lung suggests that changes in bacterial burden are disease relevant and unlikely simply secondary to the architectural distortion.

One of the many paradoxes in fibrotic lung disease is that, despite the existence of often extensive traction bronchiectasis, the productive symptoms seen in bronchiectasis rarely develop [23]. While the bacterial burden in the lower airways in IPF is magnitudes lower than that seen in cystic fibrosis or bronchiectasis, it is elevated compared with health. The quantity and composition of the respiratory microbiome is influenced by the balance between the rate of immigration and clearance. The primary routes of immigration are microaspiration and inhalation of airborne bacteria while elimination is achieved through a combination of the cough response, mucociliary clearance and the innate and adaptive immune response. In disease, the balance between these two opposing factors is lost, resulting in dysbiosis [24]. Given the strong association with GORD and IPF, microaspiration is likely to be a large driver in this cohort of patients [25]. Indeed, gastric bacteria have been identified in the respiratory microbiome supporting this [12]. However, it is not only immigration which may be abnormal in IPF, given the widespread abnormalities in the mucin genetic architecture [26–28]. The mucin glycoproteins are a major structural component of the mucus barrier, maintaining the hydration of the airway epithelium and crucially entrapping particles for removal by mucociliary clearance. In mice, *MUC5B* appears essential for normal macrophage function and effective mucociliary clearance of bacteria [29]. This leaves the hypothesis that a loss of balance between bacterial immigration and clearance results in the increased bacterial burden in the lower airways resulting in prolonged and repetitive exposure, triggering an exaggerated interstitial injury and eventually leading to the development of fibrosis.

Despite the increased microbial burden, there was no host response at a cellular level seen when examining BAL differential cell counts. There are conflicting data regarding the prognostic implications of BAL neutrophilia in IPF. The previous largest study to date demonstrated an increased risk of mortality at 12 months with increased BAL neutrophil counts, but this association was lost after a year, leading the investigators to hypothesise they may be identifying a subgroup of individuals with more active disease [30]. Here, we demonstrate the presence of increased neutrophils in the airways of patients with IPF but no association with their counts and overall bacterial burden. We also find no association between BAL cellular profiles and the bacterial burden in the airways, highlighting the difference between the stable microbiome and periods of infection, in which a cellular response is seen. This conforms with the theory that repetitive microinjuries to the epithelium trigger the disease, and you would not expect such a large cellular response. Therefore, more nuanced methods of detecting response, such as characterisation of the epithelial and airway transcriptome, may be required and should be included in any future studies.

While traction bronchiectasis and honeycombing are clearly defined and distinct entities, radiologically, they are likely to represent two aspects of the continuous spectrum of pathological remodelling that occurs in IPF. Both features have been associated with disease behaviour and clinical outcome, as has the extent of overall fibrosis [31]. There was, however, no correlation between the extent of honeycombing or traction bronchiectasis and bacterial burden, which remains a predictor of progression even accounting for these. A number of the subjects had radiology indeterminant for UIP and the multidisciplinary meeting had access to clinical data, serology and BAL cell differential counts to support the diagnosis of IPF. The relative proportions of CT UIP morphologies (definite, probable, indeterminate or inconsistent) in our cohort of MDT IPF diagnoses are similar to previously published proportions [32]. There were no links with extent of fibrosis or traction bronchiectasis, nor differences between the radiological diagnostic cohorts or probable *versus* definite UIP. We also considered if concomitant disease cofounders could have been driving this burden and given the clinical association between PPFE and infection, this was specifically interrogated. Almost one-third of the cohort had radiological evidence of PPFE and there was no difference in bacterial burden between those with and without disease or when simply considering disease extent. Although this is an interesting finding, the number of subjects with PPFE were small and this lack of association will have to be validated in larger cohorts, in which it would also be interesting to examine the microbiome in subjects with isolated PPFE.

Recently the Correlating Outcomes With Biochemical Markers to Estimate Time-progression in IPF (COMET) authors sought to examine the relationship between the microbiome and the presence or absence of honeycombing in a subgroup of 68 subjects with IPF [33]. Simply dichotomising the cohort based on the presence or absence of honeycombing they found no association with the lower airways bacterial burden. The prospective nature and quantitative radiological analysis undertaken here enabled us to build and expand upon their work. Our study design specifically allowed us to answer questions about the graded relationships between radiological change and bacterial burden. This has enabled us to undertake a far more detailed and nuanced analysis and strengthens our conclusions that the increased bacterial burden is not simply related to the extent of radiological disease.

Our work [34], and that of O'Dwyer *et al.* [33], also raises a number of issues related to the potential for antimicrobial therapies in IPF [34, 35], some of which are already being trialled (NCT02759120 and 17464641). While there is clear microbial dysbiosis in the lower airways in patients with IPF, the only consistent finding has been that of an increased bacterial burden. None of the therapies being trialled at present has ever demonstrated efficacy at reducing bacterial burden. Cautionary tales from the bronchiectasis field must be heeded, as heterogeneous responses to antibiotics have led to multiple antibiotic trials failing to meet their primary endpoints. Indeed, recently Sibila *et al.* [36] have demonstrated, in a *post hoc* analysis of multiple trials of inhaled antibiotics, that patients with higher bacterial burden consistently demonstrated a clinical response and improved quality of life. It is therefore conceivable that a more targeted approach may also be required for patients with IPF rather than simply treating indiscriminately. However, this study showed no evidence that bacterial burden can be predicted from radiology or differential cell counts.

This work has a number of limitations. First, BAL was undertaken in the right middle lobe, while radiographic extent of disease was assessed globally. This standardised approach was undertaken in line with current diagnostic and research standards; however, we appreciate an in-depth geographical survey of multiple lobes may have provided more information and should be considered in any future studies. This would also provide the perfect opportunity to assess the regional differences in both bacterial burden and microbial composition in fibrotic lung disease. This study was designed to investigate the relationship between bacterial burden and radiological disease extent and we did not undertake any characterisation of the bacterial communities. While, to date, increased bacterial burden is the most consistent microbiome-related finding in IPF this may simply reflect the ease and reproducibility of this measurement, compared with more complex 16S amplicon analysis. Larger cohorts and more standardised sequencing/analysis pipelines [37] (allowing meta-analysis) will be required to reveal more consistent relationships between specific community composition and clinical outcomes. Given the known association between the single nucleotide polymorphism rs35705950 of the *MUC5B* mucin gene [38], these prospective studies should also factor in the effect of genotype on bacterial burden. Another limitation is that radiology interpretation of CT forms a key part of the MDT, potentially confounding any study on CT findings. We attempted to minimise this impact by ensuring that at least one scoring radiologist was independent from the MDT involved in making the final diagnosis of IPF.

While our hypothesis was that that architectural destruction acts as a reservoir to increase bacterial burden, an alternate exists where the increased bacterial burden may indeed cause the architectural destruction. Our data are not supportive of either of these two scenarios but longitudinal and mechanistic studies will be required to support this. There is a known association between GORD reflux and IPF. Many patients are on PPI which have been linked to an increased risk of pneumonia [21, 39]. While we did not set out to address this issue prospectively, we demonstrated no association between GORD or PPI use and bacterial burden. Though more formal prospective studies with objective measurements of acid and non-acid reflux will be required to answer this question directly.

We demonstrate that an elevated bacterial burden predicts mortality in IPF but is independent of radiological features, differential cell counts and extent of disease. We would argue that the independent nature of this association supports a relationship with the underlying pathogenetic mechanisms. However, functional studies are urgently needed, especially as interventional studies are already enrolling and the potential for bacterial load to guide antibiotic therapy for patients with IPF should be prospectively tested. 

## Supplementary material

10.1183/13993003.01519-2019.Supp1 **Please note:** supplementary material is not edited by the Editorial Office, and is uploaded as it has been supplied by the author.
Supplementary material ERJ-01519-2019.SUPPLEMENT

## Shareable PDF

10.1183/13993003.01519-2019.Shareable1 This one-page PDF can be shared freely online.
Shareable PDF ERJ-01519-2019.Shareable
