# Supplementary material for: Bacterial burden in the lower airways predicts disease progression in idiopathic pulmonary fibrosis and is independent of radiological disease extent
Source: Eur Respir J. 2020 Apr 2;55(4):1901519. doi: 10.1183/13993003.01519-2019 (PMC7136009; doi:10.1183/13993003.01519-2019)
Supplement: Supplementary file 1 [file ERJ-01519-2019-supplement.pdf]

Bacterial burden in the lower airways predicts disease progression in idiopathic pulmonary fibrosis and is independent of radiological disease extent

Authors: Rachele Invernizzi[1*], Joseph Barnett[1,2*], Bhavin Rawal[2], Arjun Nair[3], Poonam Ghai[1], Shaun Kingston[1], Felix Chua[2], Zhe Wu[1,2], Athol U. Wells[2], Elizabeth R. Renzoni[2], Andrew G. Nicholson[1,2], Alexandra Rice[2], Clare M. Lloyd[1], Adam J. Byrne[1], Toby M. Maher[1,2], Anand Devaraj[1,2] and Philip L. Molyneaux[1,2].

[*]Contributed equally as first authors

Affiliations:

[1]National Heart and Lung Institute, Imperial College London, UK.
[2]Royal Brompton Hospital, UK.
[3]Department of Radiology, University College Hospital, London, UK.

Corresponding author: Dr Philip L. Molyneaux, Fibrosis Research Group, National Heart and Lung Institute, Imperial College London, UK. E-mail: p.molyneaux@imperial.ac.uk

ONLINE SUPPLEMENT

Results

Supplementary Figure S1. Comparison between idiopathic pulmonary fibrosis (IPF) bronchoalveolar lavage (BAL) samples (*n* = 193) and negative control samples (*n* = 12).

Supplementary Figure S2. Bronchoalveolar lavage differential cell counts.

Supplementary Figure S3. Association between total number of leukocytes in bronchoalveolar lavage (BAL) fluid and bacterial burden.

Supplementary Figure S4. Association between lung function and bronchoalveolar lavage (BAL) bacterial burden.

Supplementary Figure S5. Bronchoalveolar lavage (BAL) bacterial burden of idiopathic pulmonary fibrosis (IPF) patients with either indeterminate or probable/definite usual interstitial pneumonia (UIP).

Supplementary Table S1. Information regarding specific treatments.

Supplementary Table S2. Bronchoalveolar lavage (BAL) differential cell counts and their prediction of BAL bacterial burden.

Supplementary Table S3. Agreement between two radiologist scorers measured by Cohen's kappa with squared ratings (k) or the interclass correlation coefficient (icc).

Supplementary Figures and Tables

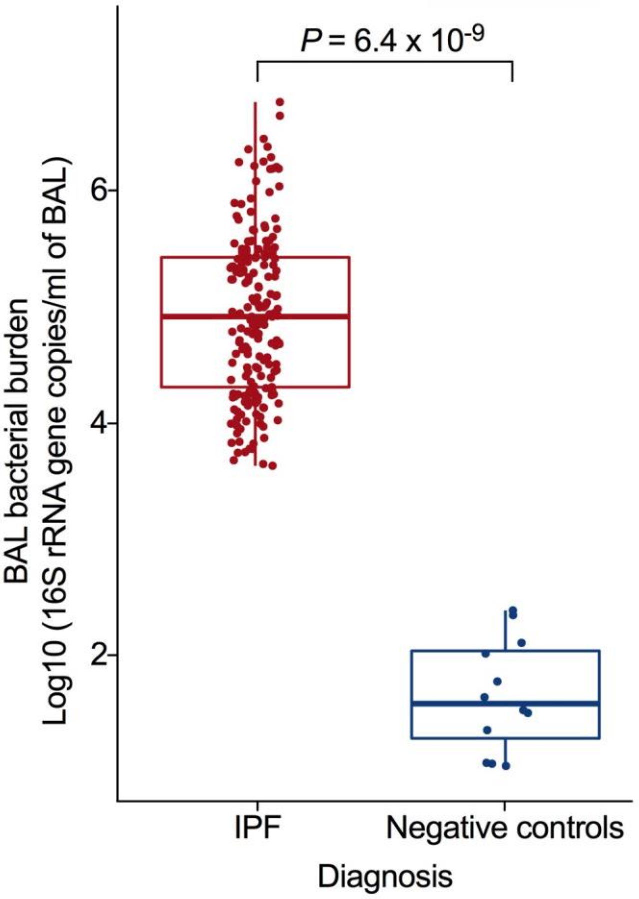

Supplementary Figure S1. Comparison between idiopathic pulmonary fibrosis (IPF) bronchoalveolar lavage (BAL) samples (*n* = 193) and negative control samples (*n* = 12). Bacterial burden calculated by qPCR and expressed as log10 16S rRNA gene copies/ml of BAL. Statistical significance tested with Mann-Whitney test.

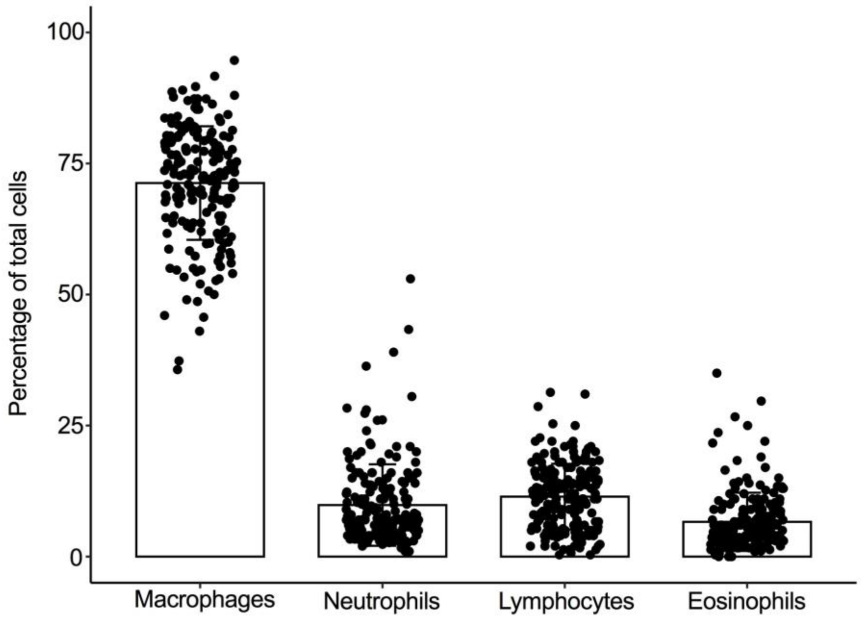

Supplementary Figure S2. Bronchoalveolar lavage differential cell counts. Cell counts of individuals with idiopathic pulmonary fibrosis (n = 193) are represented as percentage of total cells. Data are presented as mean $\pm$ standard deviation.

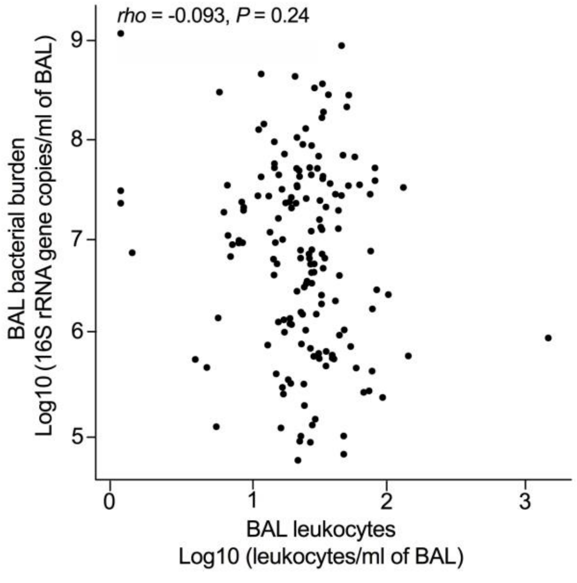

Supplementary Figure S3. Association between total number of leukocytes in bronchoalveolar lavage (BAL) fluid and bacterial burden. No correlation was observed between total number of leukocytes in BAL fluid and bacterial burden as measured by Spearman's *rho*. Bacterial burden calculated by qPCR and expressed as log10 16S rRNA gene copies/ml of BAL. Total number of leukocytes in BAL fluid were log transformed to approximate normal distribution.

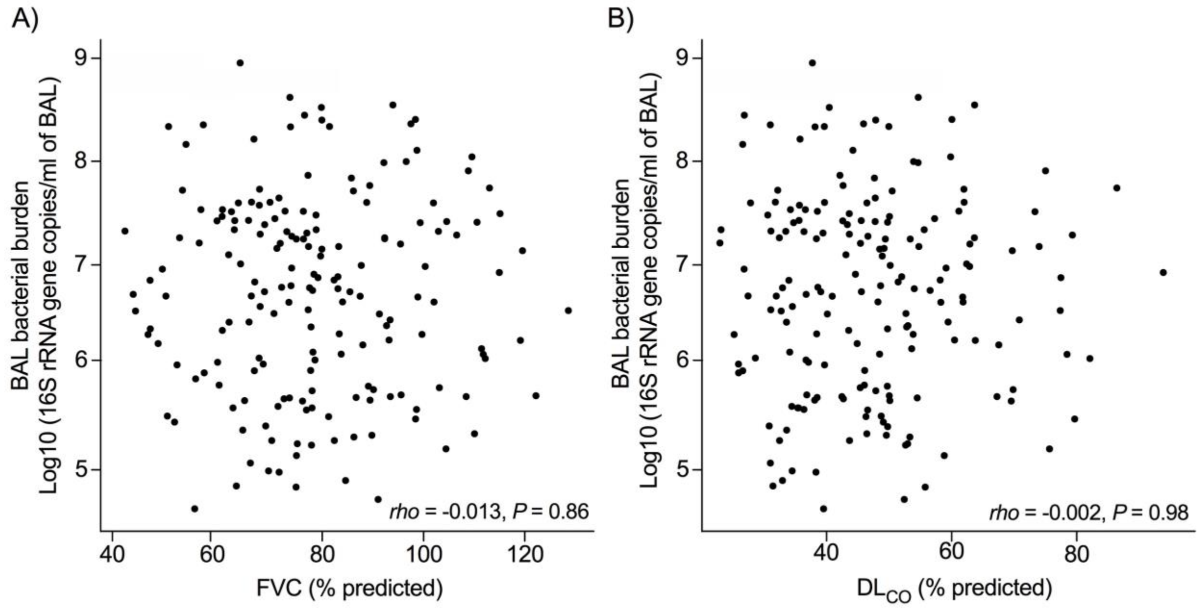

Supplementary Figure S4. Association between lung function and bronchoalveolar lavage (BAL) bacterial burden. No correlation was seen between bacterial burden and (A) FVC (% predicted) or (B) DL_{CO} (% predicted) as measured by Spearman's *rho*. Bacterial burden calculated by qPCR and expressed as log10 16S rRNA gene copies/ml of BAL. FVC: forced vital capacity, DL_{CO}: diffusing capacity of the lung for carbon monoxide.

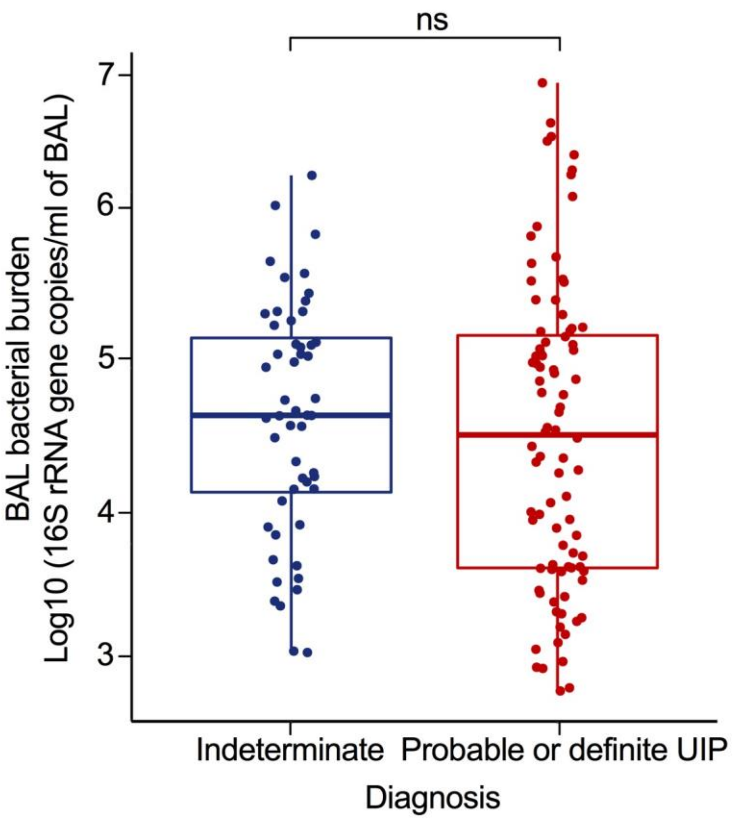

Supplementary Figure S5. Bronchoalveolar lavage (BAL) bacterial burden of idiopathic pulmonary fibrosis (IPF) patients with either indeterminate or probable/definite usual interstitial pneumonia (UIP). No differences (P = 0.328) in bacterial burden were observed when comparing IPF patients with either indeterminate or probable/definite UIP. Bacterial burden calculated by qPCR and expressed as log10 16S rRNA gene copies/ml of BAL. Statistical significance tested with Mann-Whitney test.

Supplementary Table S1. Information regarding specific treatments.

Treatment	IPF (*N* = 193)
*PPI *n* (%)	97 (91)
**Oral corticosteroids *n* (%)	21 (11)
Inhaled corticosteroids *n* (%)	36 (19)
Subsequent antifibrotic therapy *n* (%)	67 (35)

IPF: idiopathic pulmonary fibrosis; PPI: proton pump inhibitor; *PPI: 107/193 patients had concomitant gastroesophageal reflux. 97/107 patients were treated with PPI; **Steroids: 21/193 patients were administered prednisolone (<10mg/day).

Supplementary Table S2. Bronchoalveolar lavage (BAL) differential cell counts and their prediction of BAL bacterial burden. No association was seen between BAL cell differential (percentage of total cells) and bacterial burden (*n* = 193).

	β Estimate	SE	*P*-value
Macrophages	-0.149	0.011	0.882
Neutrophils	0.043	0.015	0.966
Lymphocytes	-0.076	0.019	0.940
Eosinophils	0.326	0.021	0.745

Univariate regression performed against log transformed 16S rRNA gene copy number. SE: standard error.

Supplementary Table S3. Agreement between two radiologist scorers measured by Cohen's kappa with squared ratings (k) or the interclass correlation coefficient (icc).

Variable	Agreement
Fibrosis extent (icc)	0.49
Honeycombing extent (icc)	0.75
Traction bronchiectasis extent (icc)	0.51
UIP pattern (k)	0.42
PPFE (k)	0.52

UIP: usual interstitial pneumonia; PPFE: pleuroparenchymal fibroelastosis.
