# Supplementary material for: Bacterial burden in the lower airways predicts disease progression in idiopathic pulmonary fibrosis and is independent of radiological disease extent
Source: Eur Respir J. 2020 Apr 2;55(4):1901519. doi: 10.1183/13993003.01519-2019 (PMC7136009; doi:10.1183/13993003.01519-2019)
Supplement: Supplementary file 2 [file ERJ-01519-2019-Shareable.pdf]

SHAREABLE PDF

Bacterial burden in the lower airways predicts disease progression in idiopathic pulmonary fibrosis and is independent of radiological disease extent

Rachele Invernizzi[1,4], Joseph Barnett[1,2,4], Bhavin Rawal[2], Arjun Nair[3], Poonam Ghai[1], Shaun Kingston[1], Felix Chua[2], Zhe Wu[1,2], Athol U. Wells[2], Elizabeth R. Renzoni[2], Andrew G. Nicholson[1,2], Alexandra Rice[2], Clare M. Lloyd[1], Adam J. Byrne[1], Toby M. Maher[1,2], Anand Devaraj[1,2] and Philip L. Molyneaux[1,2]

Affiliations: [1]National Heart and Lung Institute, Imperial College London, London, UK. [2]Royal Brompton Hospital, London, UK. [3]Dept of Radiology, University College Hospital, London, UK. [4]Contributed equally as first authors.

Correspondence: Philip L. Molyneaux, Fibrosis Research Group, National Heart and Lung Institute, Imperial College London, UK. E-mail: p.molyneaux@imperial.ac.uk

@ERSpublications
Elevated bacterial burden predicts mortality in IPF and is independent of both radiological features and extent of disease http://bit.ly/2RaDbdv

Cite this article as: Invernizzi R, Barnett J, Rawal B, *et al*. Bacterial burden in the lower airways predicts disease progression in idiopathic pulmonary fibrosis and is independent of radiological disease extent. *Eur Respir J* 2020; 55: 1901519 [https://doi.org/10.1183/13993003.01519-2019].

This single-page version can be shared freely online.

ABSTRACT Increasing bacterial burden in the lower airways of patients with idiopathic pulmonary fibrosis confers an increased risk of disease progression and mortality. However, it remains unclear whether this increased bacterial burden directly influences progression of fibrosis or simply reflects the magnitude of the underlying disease extent or severity.

We prospectively recruited 193 patients who underwent bronchoscopy and received a multidisciplinary diagnosis of idiopathic pulmonary fibrosis. Quantification of the total bacterial burden in bronchoalveolar lavage fluid was performed by 16S rRNA gene qPCR. Imaging was independently evaluated by two readers assigning quantitative scores for extent, severity and topography of radiographic changes and relationship of these features with bacterial burden was assessed.

Increased bacterial burden significantly associated with disease progression (HR 2.1; 95% CI 1.287–3.474; p=0.0028). Multivariate stepwise regression demonstrated no relationship between bacterial burden and radiological features or extent of disease. When specifically considering patients with definite or probable usual interstitial pneumonia there was no difference in bacterial burden between these two groups. Despite a postulated association between pleuroparenchymal fibroelastosis and clinical infection, there was no relationship between either the presence or extent of pleuroparenchymal fibroelastosis and bacterial burden.

We demonstrate that bacterial burden in the lower airways is not simply secondary to the extent of the underlying architectural destruction of the lung parenchyma seen in idiopathic pulmonary fibrosis. The independent nature of this association supports a relationship with the underlying pathogenic mechanisms and highlights the urgent need for functional studies.

Copyright ©ERS 2020. This version is distributed under the terms of the Creative Commons Attribution Licence 4.0.
